# A Granger causality analysis of groundwater patterns over a half-century

**DOI:** 10.1038/s41598-019-49278-8

**Published:** 2019-09-06

**Authors:** Nitin K. Singh, David M. Borrok

**Affiliations:** Geosciences and Geological and Petroleum Engineering, Missouri University of Science and Technology, Rolla, MO 65409 USA

**Keywords:** Environmental impact, Hydrogeology, Hydrology

## Abstract

Groundwater depletion in many areas of the world has been broadly attributed to irrigation. However, more formal, data-driven, causal mechanisms of long-term groundwater patterns have not been assessed. Here, we conducted the first Granger causality analysis to identify the “causes” of groundwater patterns using the rice-producing parishes of Louisiana, USA, as an example. Trend analysis showed a decline of up to 6 m in groundwater level over 51 years. We found that no single cause explained groundwater patterns for all parishes. Causal linkages were noted between groundwater and area harvested, number of irrigation wells, summer precipitation totals, and drought. Bi-directional linkages were noted between groundwater and rice yield, suggesting feedback between both time series. Causal linkages were absent between groundwater and many drivers where significant correlations were noted, highlighting the importance of using robust causal relationships over illusive correlations to detect the cause. These results advance our understanding of groundwater dynamics and can reveal a key connection between food and groundwater.

## Introduction

Hydrological models^1–3^ and satellite observations^4,5^ have shown rapid depletion of groundwater from regional to global scales. Besides the threat to fresh water availability, the decline in groundwater can have detrimental effects on food production^6,7^ and ecosystems and associated biodiversity that rely on groundwater for survival^8–10^. Rising interannual climatic variability and increasing food demand due to rising human population are likely to intensify the stress on groundwater resources. Using hydrological and crop models, studies have linked food production to groundwater depletion from regional to global scales^11–13^. Given the intimate connection between groundwater and food security^14^, there is a need to understand the major drivers of groundwater patterns in agricultural regions to meet food supply requirements without jeopardizing the limited groundwater resources.

Several studies have analyzed long-term (>30 years) observations of groundwater levels^15–17^, but few of these have focused on agricultural regions impacted by pumping^18,19^. In many of these studies, groundwater decline could be correlated with climatic drivers such as precipitation, temperature, drought, and agricultural drivers such as pumping. However, these correlation-derived relationships alone should not be relied upon to establish cause and effect relationships. Correlation is innately embedded in our thinking when it comes to determining the relationship between two variables. However, in a complex, nonlinear environmental system, correlations can be an illusion^20^ and may not detect the true nature of the relationships between the drivers and groundwater patterns. In other words, correlation does not indicate causation, and the absence of correlation does not necessarily mean there is no causation. There remains a critical need to advance our knowledge of “causes” of groundwater patterns while making use of data-driven, robust statistical approaches such as causality analysis^21,22^.

One of the most robust methods available to detect the causal linkages between two-time series is Granger Causality analysis^21^ [see Methods section for details]. Initially, Granger Causality was introduced in the field of economics. Due to the robust nature and the flexibility of the Granger Causality^23^, it has been widely used in many climate change attribution studies^22,24,25^. In addition to revealing the causal linkages, Granger Causality can be useful for determining the direction of causal linkages between variables^18^. After several decades of research, the implementation of Granger Causality to advance our understanding of cause and effect relationships of groundwater patterns remains unexplored.

In this study, we conduct the first data-driven, Granger causality analysis to identify the “causes” of groundwater patterns at multi-decadal scales. For this test case, we implemented the causality analysis in the state of Louisiana, USA, which in some locations is under immense groundwater stress due to intensive agricultural activities^26^. The state of Louisiana also serves as an ideal test bed for this analysis because it possesses a rich and a unique combination of climatic and agricultural time series recorded at annual time-scales over 51 years. The objective of this study was to identify any causal linkages between groundwater level patterns and climatic and agricultural variables from 1965 to 2015. The work was conducted in the state of Louisiana, as an example, but the proposed approach can be adapted to other agricultural regions of the world to reveal causal mechanisms influencing groundwater patterns.

## Results

### Trend analysis

**Figure 1:**
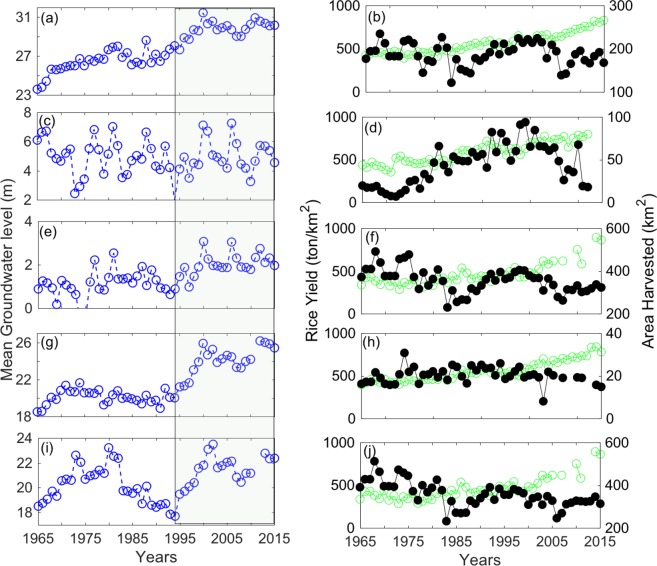
Time series of mean groundwater level, rice yield and area harvested for five parishes: EV (**a**,**b**), EC (**c**,**d**), SM (**e**,**f**), AC (**g**,**h**), and JD (**i**,**j**) of Louisiana. Open blue circles represent mean groundwater level, open green circles represent rice yield and filled black circles represent harvested area for rice. Shaded rectangle shows the duration in which most of the wells showed steep decline in groundwater level. The water level is measured from the land surface so higher the number drier the well.

**Figure 2:**
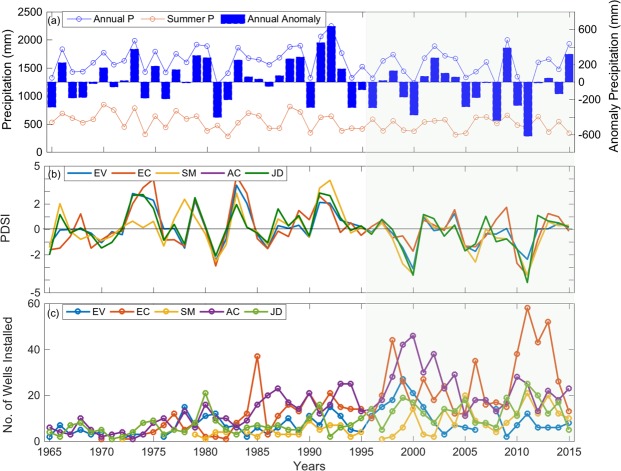
Annual and summer precipitation totals and annual precipitation anomalies for a climate station in the southern part of the state (**a**), Palmer drought severity index (PDSI) (**b**), and number of irrigation wells installed (**c**) for five parishes of Louisiana. Shaded green area shows the period with high frequency of droughts based on negative PDSI values and annual precipitation anomalies since 1995.

Groundwater levels exhibited significant declines over the last 51 years ranging from 1.5–6 m in EV, SM and AC parishes (P < 0.05; Table 1, Fig. 1). The well in EC (the Mississippi River Alluvial aquifer) was the shallowest among all parishes and showed highly variable temporal patterns with no significant trend over the 51 year period. All of the remaining wells (in the Chicot aquifer) showed a steep decline in water level post-1995. Rice yield showed a significant increase in all parishes with the variable rate of increase (P < 0.05, Table 1). Area harvested increased in EC, declined in JD, or showed no trends in EV, AC, and SM parishes. The number of wells installed for irrigation over time showed upward trends in all parishes (P < 0.05, Table 1), and the abrupt rise in well counts corresponded to highly negative Palmer Drought Severity Index (PDSI) values, especially post-1995 (Fig. 2). Summer precipitation totals declined by 157 mm, and the minimum air temperatures increased substantially, during 51 years for a climate station in the southern part of the state (Table 1). No significant unidirectional trends were detected for the rest of the datasets for any parish (P > 0.05).

**Table 1:** Sen Slope (P values) estimated during the study period^ϒ^.

Parishes	Mean GW Stage (m/year)	Rice Yield (ton/km^2^/yr)	Area Harvested (km^2^/year)	Summer Precipitation (mm/yr)	Irrigation Wells Installed	T_min_ (°C/yr)
EV	0.11 (<0.001)	7.36 (<0.001)	−0.169 (0.50)	−3.08 (0.01)	0.09 (0.69)	0.02 (0.004)
EC	−0.005 (0.81)	8.21 (<0.001)	1.21 (0.01)	3.81 (0.78)	0.61 (<0.001)	−0.01 (0.29)
SM	0.02 (<0.001)	6.44 (<0.001)	0 (0.58)	−3.08 (0.01)	0.25 (<0.001)	0.02 (0.004)
AC	0.12 (0.02)	7.20 (<0.001)	−1.76 (0.06)	−3.08 (0.01)	0.44 (0.001)	0.02 (0.004)
JD	0.03 (0.58)	6.69 (<0.001)	−2.24 (0.002)	−3.08 (0.01)	0.21 (0.03)	0.02 (0.004)

^ϒ^Datasets that showed significant (P < 0.05) trends for any parish are shown here.

### Granger causality

Phillps-Perron test showed that most of the time series were stationary for the study period (Table S1). Summer precipitation totals, PDSI, area harvested, and the number of irrigation wells were found to Granger cause groundwater response (P < 0.05, Table 2). However, the causal linkages varied among parishes and no common cause was noted in all parishes (Table 2). A relatively weak Granger causality was noted between groundwater and rice yield and annual precipitation totals (P ≦ 0.11). Further, we found that groundwater levels can also Granger cause rice yield, indicating potential feedback between both time series (Table 2).

**Table 2:** Granger Causality analysis for the hypotheses tested in the study.

Granger Causality F stat (P Value)	Parishes				
Null Hypotheses	EV	EC	SM	AC	JD
Annual Precipitation does not GC GW	1.81 (0.09)	0.61 (0.54)	0.63 (0.53)	2.32 (0.1)	0.37 (0.54)
Summer Precipitation does not GC GW	0.95 (0.41)	2.4 (0.09)	1.43 (0.24)	0 (0.98)	3.08 (0.03)
T Min does not GC GW	1.43 (0.23)	0.87 (0.41)	1.93 (0.16)	1.08 (0.30)	0.29 (0.59)
Tmax does not GC GW	0.29 (0.74)	0.94 (0.39)	0.50 (0.67)	0.47 (0.49)	0.08 (0.76)
PDSI does not GC GW	1.23 (0.29)	0.5 (0.67)	7.55 (0.007)	0.44 (0.50)	0.11 (0.73)
GDD does not GC GW	0.33 (0.71)	2.22 (0.11)	0.90 (0.34)	0.45 (0.5)	0.86 (0.35)
Irrigation wells does not GC GW	1.45 (0.19)	0.91 (0.34)	1.59 (0.20)	3.09 (0.006)	2.24 (0.13)
Rice yield does not GC GW	1.71 (0.11)	1.71 (0.11)	0.04 (0.83)	1.1(0.38)	0.04 (0.82)
GW does not GC Rice yield	1.41 (0.21)	2.21 (0.03)	0.01 (0.89)	2.35 (0.02)	0.04 (0.83)
Area harvested does not GC GW	3.41 (0.003)	3.12 (0.006)	0.16 (0.68)	0.80 (0.62)	0.31 (0.57)

### Correlations

**Figure 3:**
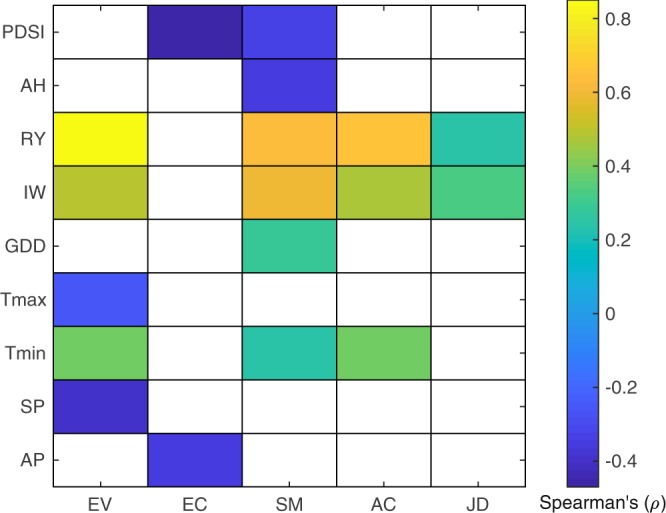
Spearman’s correlation (ρ) between groundwater levels and climatic and agriculture drivers for the five parishes (EV, EC, SM, AC, JD) over 51 years. Shaded color represents significant (P < 0.05) correlations; white blanks represents non-significant (P > 0.05) relationships. Abbreviations- Parishes: Evangeline (EV), East Carroll (EC), St Martin (SM), Acadia (AC), Jefferson Davis (JD); Annual Precipitation totals (AP), Summer Precipitation totals (SP), Minimum Temperature (T_min_), Maximum Temperature (T_max_), Palmer Drought Severity Index (PDSI), Growing Degree Days (GDD), Rice Yield (RY), Irrigation Wells (IW), Area Harvested (AH).

**Figure 4:**
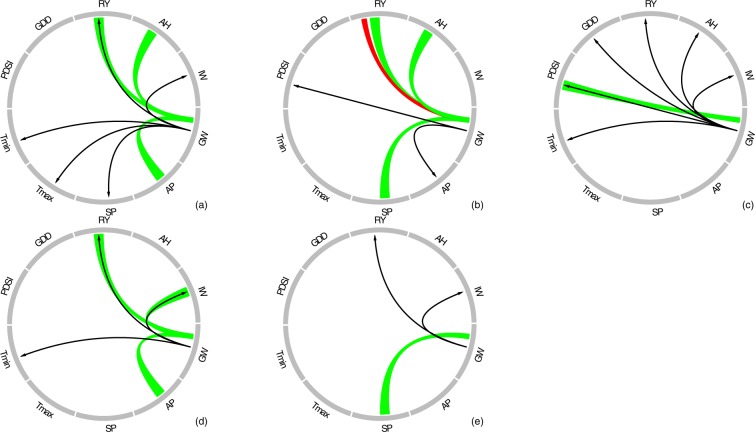
Summary of causal and correlation derived relationships estimated between groundwater levels and agricultural and climatic variables for all parishes: Evangeline (**a**), East Carroll (**b**), St. Martin (**c**), Acadia (**d**), and Jefferson Davis (**e**). Green ribbon represents causal linkages between groundwater levels and agricultural and climatic variables. Red ribbon represents bi-directional causal linkages between groundwater levels and rice yield. Black arrow represents correlation between groundwater levels and agricultural and climatic variables. There are only four instances in which causal and correlation derived relationships are in agreement. Variables that did not show either causal or correlations relationships in any parish are not shown here. Abbreviations- Groundwater Level (GW), Annual Precipitation totals (AP), Summer Precipitation totals (SP), Minimum Temperature (T_min_), Maximum Temperature (T_max_), Palmer Drought Severity Index (PDSI), Growing Degree Days (GDD), Rice Yield (RY), Irrigation Wells (IW), Area Harvested (AH).

Spearman’s correlations (ρ) were found between groundwater and many of the climatic and agricultural drivers for all parishes (P < 0.05; Fig. 3). For instance, rice yield and the number of irrigation wells showed significant relationships with groundwater levels for most of the parishes (P < 0.05; Fig. 3). Minimum air temperature also exhibited correlations with groundwater level for several parishes (P < 0.05; Fig. 3). The PDSI in SM parish and precipitation totals in EC parish were the only climatic variables where correlation derived relationships were consistent with the causal linkages. Figure 4 summarizes all correlations and causal derived relationships for all parishes.

## Discussion

Several water budgeting and hydrological modeling studies have broadly attributed groundwater patterns to irrigation withdrawals^2,27^. Some of these modeling studies have simulated the effect of crop production on irrigation demand and subsequently groundwater storage^28–30^. Our data-driven, causality analysis highlighted a range of other causal relationships that can contribute to groundwater decline besides irrigation. Further, we found a relatively weak bi-directional relationship between groundwater levels and rice yield during the study period.

The causal linkages were noted between groundwater levels and drought (i.e., PDSI) and the number of irrigation wells. For instance, the PDSI and precipitation anomalies suggest that the state of Louisiana experienced several droughts post-1995 (Fig. 2). To offset the droughts, farmers dramatically increased installation of irrigation wells and relied more heavily on groundwater resources (Fig. 2). This in-turn resulted in steep decline in groundwater level but had almost no effect on rice yield (Fig. 1).

As water availability plays a significant role in rice production, it is likely that the availability of groundwater may Granger cause rice yield over the long-term (Fig. 4). On the other hand, higher rice production in the past can encourage farmers to withdraw more groundwater suggesting that rice yield can also cause groundwater level changes as is evident in EC parish (Fig. 4). These results are in agreement with a recent modeling study that used rice production, as one of the predictors, to simulate groundwater levels in India^31^ which is one of the leading rice producing countries in the world. Together, bi-directional causal linkages noted in our work between groundwater and rice yield suggest that both time series may co-evolve in time. However, additional work is needed to test whether this relationship holds at larger spatial scales and in other study locations. Subsequently, correlation analysis showed statistically significant relationships between groundwater and rice yield for four parishes despite no causal relationships in some cases. This illustrates how conflating correlations with cause and effect relationships can be misleading (Fig. 4).

The areas of rice harvested showed causal linkages with groundwater levels for some of the parishes such as EC (Fig. 4). The area harvested for the other parishes remain unchanged or even declined, but groundwater levels continued to decline, demonstrating that the decline in groundwater is not necessarily sensitive to the amounts of cropped areas in this region. These results pointed to the dominance of climate-induced drought over agricultural-related causes in influencing the patterns of groundwater. However, it is worth mentioning that increases in the cropped area can lead to a higher demand for irrigation, resulting in groundwater depletion. Overall, these findings highlight the need for careful evaluation of the relative importance of causal mechanisms that may influence groundwater patterns.

Using hydrological models, studies have overwhelmingly attributed groundwater decline to excessive withdrawal due to irrigation^27^. Our work suggests that attributing groundwater depletion patterns solely to an individual agricultural driver may be an oversimplification of a complex problem. The relative importance of the drivers may change substantially even over small spatial scales (e.g., parishes). We also recognize that there are many additional agricultural and socio-economic drivers that may influence groundwater patterns that could not be analyzed here due to the lack of long-term data availability.

Several studies have shown significant correlations between groundwater and precipitation and temperature^15,16,30,32^. In general, we would expect linkages between groundwater and precipitation, because greater precipitation totals typically lead to more groundwater recharge. Our findings suggest strong causal linkages between groundwater and annual precipitation totals, particularly so for annual summer precipitation totals (Table 2). This strong causal relationship can be attributed to the timing of summer precipitation, which overlaps with the growing season of rice and provides much needed additional moisture for rice. The significant declining trend in summer precipitation noted for the southern part of the study region can put additional stress on the groundwater (Table 1). On the other hand, an increase in air temperature can lead to higher evapotranspiration resulting in depletion of groundwater levels. Our findings indicated significant correlations between groundwater and temperature (T_min_ or T_max_) for most of the parishes (Fig. 3), but no causal linkages were noted in any parish (Fig. 4). Most of these significant correlations were found merely because both groundwater level and temperature showed unidirectional trends with time. These results further illustrate that correlation can be misleading, as they do not necessarily imply causation^20^. Overall, our findings highlight the complexity of untangling the causes of groundwater patterns where climate and agriculture drivers might interact in multiple ways to influence groundwater patterns.

We conducted the Granger Causality analysis to reveal the causal linkages between groundwater and agricultural and climatic drivers. The causality analysis showed that no one “cause” could be attributed for the groundwater patterns and that causes varied even between adjacent parishes. These results highlight the importance of other climatic and agricultural drivers for groundwater patterns and the complexity in simulating groundwater levels via process-based or physical models. We found that droughts did not negatively impact rice yield in any of the parishes, and farmers relied on groundwater by dramatically increasing the number of irrigation wells, resulting in severe groundwater depletion in some parishes. In addition to pumping, other agricultural causes such as rice yield and area harvested could play significant roles in mediating the groundwater levels in the region. However, for some parishes, area harvested remained unchanged or declined, but groundwater continued to decline, pointing to the dominance of climatic over agricultural drivers. There were only four occasions when causal linkages overlapped with the correlation derived relationships, indicating that we must exercise caution while using correlations alone to detect the causes of the groundwater patterns. The novelty of this work lies in revealing in-depth insights into the causation of groundwater depletion and suggesting a robust approach to understand the intimate connections between food and groundwater. Such advancements in our understanding can ultimately lead to better management of groundwater while meeting global food demands.

## Methods

### Study sites

The state of Louisiana is the third largest producer of rice in the US^33^ and due in part to rice irrigation, a major part of the state of Louisiana is under groundwater stress^34^. There are more than a dozen rice-producing parishes (i.e., counties) in Louisiana. However, due to the gaps in long-term datasets, we identified only five parishes that had the necessary data records for the range of time series used in the study (Fig. S1). The parishes that were considered in the study include Evangeline (EV), East Carroll (EC), St. Martin (SM), Acadia (AC), and Jefferson Davis (JD).

### Datasets

Groundwater levels (depth from the land surface) were obtained from the USGS groundwater database^35^. A single representative well with almost no missing annual water level data from 1965–2015 was selected for the analysis within each parish. The study wells varied in depth from 35 m (EC), 62 m (AC), 70 m (EV), 97 m (JD), to 114 m (SM) and were located in the Chicot aquifer^36^ (e.g., EV, AC, JD, SM) and Mississippi River Valley Alluvial aquifer^37^ (e.g., EC) systems. These two aquifers are the major sources of groundwater used for irrigation in the state of Louisiana^38^.

Table 3 summarizes all raw and derived time series with their corresponding sources and methods used for computation. Two representative climate stations were used for north (EC) and south (AC, EV, SM, JD) parishes. Climatic datasets included precipitation totals (annual and summer), minimum and maximum air temperatures, Growing Degree Days (GDD), and Palmer Drought Severity Index (PDSI). The Growing Degree Days (GDD) is one of the commonly used climate indices that may exert a strong influence on crop production^39^, and it is estimated as mean air temperature above a certain threshold accumulated over time (Table 3). The Palmer Drought Severity Index (PDSI) is a climate index representative of dryness of landscape derived from a combination of variables such as precipitation, air temperature and soil moisture^40^ (Table 3). Agricultural datasets included, annual rice yield, annual area harvested, and the number of wells installed for irrigation from 1965 to 2015 for the five parishes. All climatic and agricultural variables were aggregated at an annual scale for each parish. Groundwater withdrawal for irrigation is a well-established cause of groundwater decline^18^. But the datasets for groundwater demand in Louisiana are available through USGS only at 5-year intervals resulting in less than 10 observations for the study period. To address the data limitation issue, we used the number of irrigation wells installed, which was available at an annual scale, as a surrogate for changes in groundwater demand.

**Table 3:** Climatic and agricultural variables used in the study.

Variables	Explanation of Variables	Source	Spatial Scale
Annual Precipitation totals (mm)	Daily precipitation depths were aggregated at annual scale	NOAA	North climate station for (EC Parish) and South climate station (EV, JD, SM, AC Parishes)
Summer Precipitation totals (mm)	Daily precipitation depths were aggregated for July-Oct, period critical for rice production	NOAA	North climate station for (EC Parish) and South climate station (EV, JD, SM, AC Parishes)
Minimum Air Temperature (T_min_, °C)	Daily minimum air temperatures were averaged at annual scale	NOAA	North climate station for (EC Parish) and South climate station (EV, JD, SM, AC Parishes)
Maximum Air Temperature (T_max_, °C)	Daily maximum air temperatures were averaged at annual scale	NOAA	North climate station for (EC Parish) and South climate station (EV, JD, SM, AC Parishes)
Growing Degree Days (GDD, °C)	Estimated from mean air temperature and base temp (30°C for Rice)^39^		North climate station for (EC Parish) and South climate station (EV, JD, SM, AC Parishes)
Palmer Drought Severity Index (PDSI)	Proxy for drought^40^	NOAA	Parish scale
Rice Yield (ton/km^2^)	Total rice produced per unit area	NASS	Parish scale
Area Harvested (km^2^)	Total rice area harvested	NASS	Parish scale
Number of Irrigation wells	No. of wells installed for irrigation	DNR, LA	Parish Scale

### Trend analysis

Two-tailed Mann-Kendall (MK) tests were conducted to detect significant trends at an annual scale for all datasets over the tested 51 years^41,42^. The MK test is a rank based, non-parametric method and has been widely used for determining trends in hydro-climatic datasets^43–45^. Studies show that serial auto-correlation in time series can falsely lead to a detection of a trend in MK test^46,47^. To address this issue, a pre-whitening method was implemented before detecting the trend^48^. The Sen Slope was estimated to quantify the rate of change in time series when the significant (P < 0.05) trend was detected^49^. Trend analysis was conducted in R 2.5.1^50^ using package ‘ZYP’^51^.

### Granger causality

Granger^21^ proposed causality analysis to gain a more nuanced understanding of causal linkages between two variables. In general, causality analysis relies on “cause” and “effect” relationship and makes use of the concept of predictability rather than the simple correlation between the response and the associated driver or cause^20,52^. Granger causality^21^ occurs if “our ability to predict future response of Y increases by including all information available except the current value of X” (Eq. 1). In this case variable X is said to *Granger cause* Y. “Feedback” between cause and response is established when X can Granger cause Y and Y can Granger cause X. To conduct causality analysis, Granger proposed a bi-variate model (e.g., Eqs 1 and 2) between two stationary time series (X and Y).

1 $${Y}_{t}=\mathop{\sum }\limits_{j=1}^{m}\,{a}_{j}{Y}_{t-j}+\mathop{\sum }\limits_{j=1}^{m}\,{b}_{j}{X}_{t-j}+{\varepsilon }_{t}$$

2 $${X}_{t}=\mathop{\sum }\limits_{j=1}^{m}\,{c}_{j}{X}_{t-j}+\mathop{\sum }\limits_{j=1}^{m}\,{d}_{j}{Y}_{t-j}+{\eta }_{t}$$

where, X and Y are two stationary time series; a, b, c, and d are coefficients; and ε and η are white noise. For X to Granger cause Y, b_j_ ≠ 0 in Eq. 1; for Feedback between X and Y, d_j_ ≠ 0 in Eq. 2.

Causality analysis was implemented in the following steps. First, the stationarity of each time series was tested via a non-parametric Phillips-Perron test^53^. If time series was non-stationary, differencing was implemented and vector autoregressive models were developed with the modified time series^22^. Second, vector autoregressive models were developed between “response (Y)” i.e., groundwater level and “cause (X)” i.e., each climatic and agricultural variable separately for all parishes, resulting in almost 50 vector autoregressive models. Third, two-tailed F tests were conducted to test the Null hypothesis (H_o_: X does not Granger cause Y) for the Granger causality.

Phillips-Perron test was conducted at the max lag (as shown in Eq. 3) in R 2.5.1^50^ and the outputs include, Dickey-Fuller statistics with P values. If time series was non-stationary, differencing was implemented and vector autoregressive models were developed with the modified time series as recommended^22^. Some of the major vector autoregressive model parameters included, type of regressor (i.e., trend and constant), lag order, and max lag. The lag order was automatically selected by the model based on Akaike Information Criterion (AIC), a commonly used model selection approach^54^. Max lag, the upper limit of the lag for the vector autoregressive model, was estimated^55^ as shown in Eq. (3):

3 $$Max\,Lag=12{(\frac{T}{100})}^{\frac{1}{4}}$$

where T is the length of time series. Lastly, conventional rank-based, non-parametric, Spearman’s correlation (ρ) was estimated between groundwater levels and all climatic and agricultural variables^56^. The causality analysis was performed in R 2.5.1^50^ using ‘VAR’ package^57^. Causality and correlation results were plotted in R 2.5.1^50^ using ‘Circlize’ package^58^.

## Supplementary information

Supplementary Material

## Data Availability

The datasets used in the study are publicly available from NOAA, NASS, USGS and Louisiana DNR.

## References

[1] Wada Y (2010). Global depletion of groundwater resources. Geophysical Research Letters..

[2] Scanlon BR (2012). Groundwater depletion and sustainability of irrigation in the US High Plains and Central Valley. Proceedings of the national academy of sciences..

[3] Ashraf B (2017). Quantifying anthropogenic stress on groundwater resources. Scientific reports..

[4] Rodell M, Velicogna I, Famiglietti JS (2009). Satellite-based estimates of groundwater depletion in India. Nature..

[5] Famiglietti JS (2014). The global groundwater crisis. Nature Climate Change..

[6] Jury WA, Vaux H (2005). The role of science in solving the world’s emerging water problems. Proceedings of the National Academy of Sciences..

[7] Shah, T. *et al*. Groundwater: a global assessment of scale and significance. In: Molden D (ed) Water for Food, Water for Life, International Water Management Institute (IMWI) and Earthscan, London, 395–423 (2007).

[8] Kløve B (2011). Groundwater dependent ecosystems. Part I: Hydroecological status and trends. Environmental Science & Policy..

[9] Mukherjee A, Bhanja SN, Wada Y (2018). Groundwater depletion causing reduction of baseflow triggering Ganges river summer drying. Scientific reports..

[10] Qiu J (2019). Nonlinear groundwater influence on biophysical indicators of ecosystem services. Nature Sustainability..

[11] Hanasaki N, Inuzuka T, Kanae S, Oki T (2010). An estimation of global virtual water flow and sources of water withdrawal for major crops and livestock products using a global hydrological model. Journal of Hydrology..

[12] Grafton RQ, Williams J, Jiang Q (2017). Possible pathways and tensions in the food and water nexus. Earth’s Future..

[13] Okada M (2018). Varying Benefits of Irrigation Expansion for Crop Production Under a Changing Climate and Competitive Water Use Among Crops. Earth’s Future..

[14] Turral, H., Burke, J. J. & Faurès, J. M. Climate change, water and food security. Rome: Food and Agriculture Organization of the United Nations (2011).

[15] Chen Z, Grasby SE, Osadetz KG (2004). Relation between climate variability and groundwater levels in the upper carbonate aquifer, southern Manitoba, Canada. Journal of Hydrology..

[16] Weider, K. & Boutt, D. F. Heterogeneous water table response to climate revealed by 60 years of ground water data. *Geophysical Research Letters*. **37**(24) (2010).

[17] Chaudhuri S, Ale S (2014). Long-term (1930–2010) trends in groundwater levels in Texas: influences of soils, landcover and water use. Science of the Total Environment..

[18] Russo TA, Lall U (2017). Depletion and response of deep groundwater to climate-induced pumping variability. Nature Geoscience..

[19] Hodgkins GA, Dudley RW, Nielsen MG, Renard B, Qi SL (2017). Groundwater-level trends in the US glacial aquifer system, 1964–2013. Journal of hydrology..

[20] Sugihara G (2012). Detecting causality in complex ecosystems. Science..

[21] Granger C (1969). Investigating causal relations by econometric models and cross-spectral methods. Econometrica..

[22] Zhang DD (2011). The causality analysis of climate change and large-scale human crisis. Proceedings of the National Academy of Sciences..

[23] Toda HY, Yamamoto T (1995). Statistical inference in vector autoregressions with possibly integrated processes. Journal of Econometrics..

[24] Elsner JB (2006). Evidence in support of the climate change–Atlantic hurricane hypothesis. Geophysical Research Letters..

[25] Sun Q, Miao C, AghaKouchak A, Duan Q (2016). Century‐scale causal relationships between global dry/wet conditions and the state of the Pacific and Atlantic Oceans. Geophysical Research Letters.

[26] Eldardiry H, Habib E, Borrok DM (2016). Small‐Scale Catchment Analysis of Water Stress in Wet Regions of the U.S., an Example from Louisiana. Environmental Research Letters..

[27] Siebert S (2010). Groundwater use for irrigation–a global inventory. Hydrology and Earth System Sciences..

[28] Siebert S, Döll P (2010). Quantifying blue and green virtual water contents in global crop production as well as potential production losses without irrigation. Journal of Hydrology.

[29] Dalin C, Wada Y, Kastner T, Puma MJ (2017). Groundwater depletion embedded in international food trade. Nature..

[30] Shanley JB, Chalmers AT, Mack TJ, Smith TE, Harte PT (2016). Groundwater Level Trends and Drivers in Two Northern New England Glacial Aquifers. Journal of the American Water Resources Association (JAWRA)..

[31] Bhargava A (2018). Climate variability, rice production and groundwater depletion in India. Environmental Research Letters.

[32] Dudley RW, Hodgkins GA, Nielsen MG, Qi SL (2018). Estimating historical groundwater levels based on relations with hydrologic and meteorological variables in the US glacial aquifer system. Journal of Hydrology..

[33] NASS National Agriculture Statistics Service, https://www.nass.usda.gov (accessed Oct, 2018).

[34] Borrok DM, Chen J, Eldardiry H, Habib EA (2018). Framework for Incorporating the Impact of Water Quality on Water Supply Stress: An Example from Louisiana, USA. JAWRA Journal of the American Water Resources Association..

[35] USGS United States Geological Survey, https://waterdata.usgs.gov/nwis/gw (accessed Oct, 2018).

[36] Borrok DM, Broussard WP (2016). Long-term geochemical evaluation of the coastal Chicot aquifer system, Louisiana, USA. Journal of Hydrology..

[37] Carlson, D. Systematic variability of hydraulic conductivity within the Mississippi River alluvial aquifer in northeastern Louisiana (2006).

[38] Sargent, B. P. Water use in Louisiana, 2000: Louisiana Department of Transportation and Development, Water Resources Special Report No. 15, 133 (2002).

[39] Zhu X, Troy TJ (2018). Agriculturally relevant climate extremes and their trends in the world’s major growing regions. Earth’s Future.

[40] Palmer, W. C. Meteorological drought, 30, 1–58. Washington, DC: US Department of Commerce, Weather Bureau (1965).

[41] Mann HB (1945). Non-parametric tests against trend. Econometrica..

[42] Kendall, M. G. *Rank Correlation Methods*, 4th edition, Charles Griffin, London (1975).

[43] Lettenmaier DP, Wood EF, Wallis JR (1994). Hydro-climatological trends in the continental United States, 1948–88. Journal of Climate..

[44] Borrok DM, Engle MA (2014). The role of climate in increasing salt loads in dryland rivers. Journal of Arid Environments..

[45] Singh NK (2016). Hydro-climatological influences on long-term dissolved organic carbon in a mountain stream of the southeastern United States. Journal of environmental quality..

[46] Kulkarni A, von Storch H (1995). Monte Carlo experiments on the effect of serial correlation on the Mann–Kendall test of trend. Meteorologische Zeitschrift.

[47] Hamed KH, Rao AR (1998). A modified Mann–Kendall trend test for autocorrelated data. Journal of Hydrology..

[48] Yue S, Pilon P, Phinney B, Cavadias G (2002). The influence of autocorrelation on the ability to detect trend in hydrological series. Hydrological Processes.

[49] Sen PK (1968). Estimates of the regression coefficient based on Kendall’s tau. Journal of the American Statistical Association..

[50] R Core Team, R: A Language and Environment for Statistical Computing. R Foundation for Statistical Computing, Vienna, https://www.R-project.org (2018).

[51] Bronaugh, D. & Werner, A. zyp: Zhang + Yue-Pilon trends package. R package version 0.10-1, https://CRAN.R-project.org/package=zyp (2013).

[52] Faybishenko B (2017). Detecting dynamic causal inference in nonlinear two-phase fracture flow. Advances in water resources..

[53] Phillips PC, Perron P (1988). Testing for a unit root in time series regression. Biometrika.

[54] Akaike H (1983). Information measures and model selection. Bulletin of International Statistical Institute..

[55] Schwert GW (1989). Test for Unit Roots: A Monte Carlo Investigation. Journal of Business & Economic Statistics.

[56] Spearman C (1904). The Proof and Measurement of Association between Two Things. American journal of Psychology..

[57] Pfaff, B. VAR, SVAR & SVEC Models: Implementation Within R Package vars. *Journal of Statistical Software*. **27**(4) (2008).

[58] Gu Z (2014). Circlize implements and enhances circular visualization in R. Bioinformatics..

