# Supplementary material for: A Granger causality analysis of groundwater patterns over a half-century
Source: Sci Rep. 2019 Sep 6;9:12828. doi: 10.1038/s41598-019-49278-8 (PMC6731282; doi:10.1038/s41598-019-49278-8)
Supplement: Supplementary file 1 — Supplementary Material [file 41598_2019_49278_MOESM1_ESM.pdf]

Scientific Reports

Supporting Information for

[A Granger causality analysis of groundwater patterns over a half-century]

[Nitin K Singh[1], David M Borrok[1]]

[1]Geosciences and Geological and Petroleum Engineering, Missouri University of Science and Technology, Rolla, MO 65409

Table S1 Dickey-Fuller (DF) statistics for the Phillips-Perron Test for all datasets

	Parishes				
DF stats at $P<0.05$	EV	EC	SM	AC	JD
Groundwater level	-3.89	-4.41	-4.36	-10.78§	-8.08§
Annual Precipitation	-7.33	-6.91	-7.33	-7.33	-7.33
Summer Precipitation	-8.11	-6.66	-8.11	-8.11	-8.11
T_{min}	-5.22	-5.63	-5.22	-5.22	-5.22
T_{max}	-5.47	-5.43	-5.47	-5.47	-5.47
PDSI	-5.48	-5.11	-4.89	-6.19	-6.19
GDD	-9	-5.76	-9	-9	-9
Irr. wells	-3.75	-4.91	-6.06	-3.63	-5.93
Rice Yield	-12.6	-4.21	-3.56	-14.71§	-21.54§
Area Harvested	-3.94	-13.6§	-5.69	-3.64	-3.99

§after first differencing

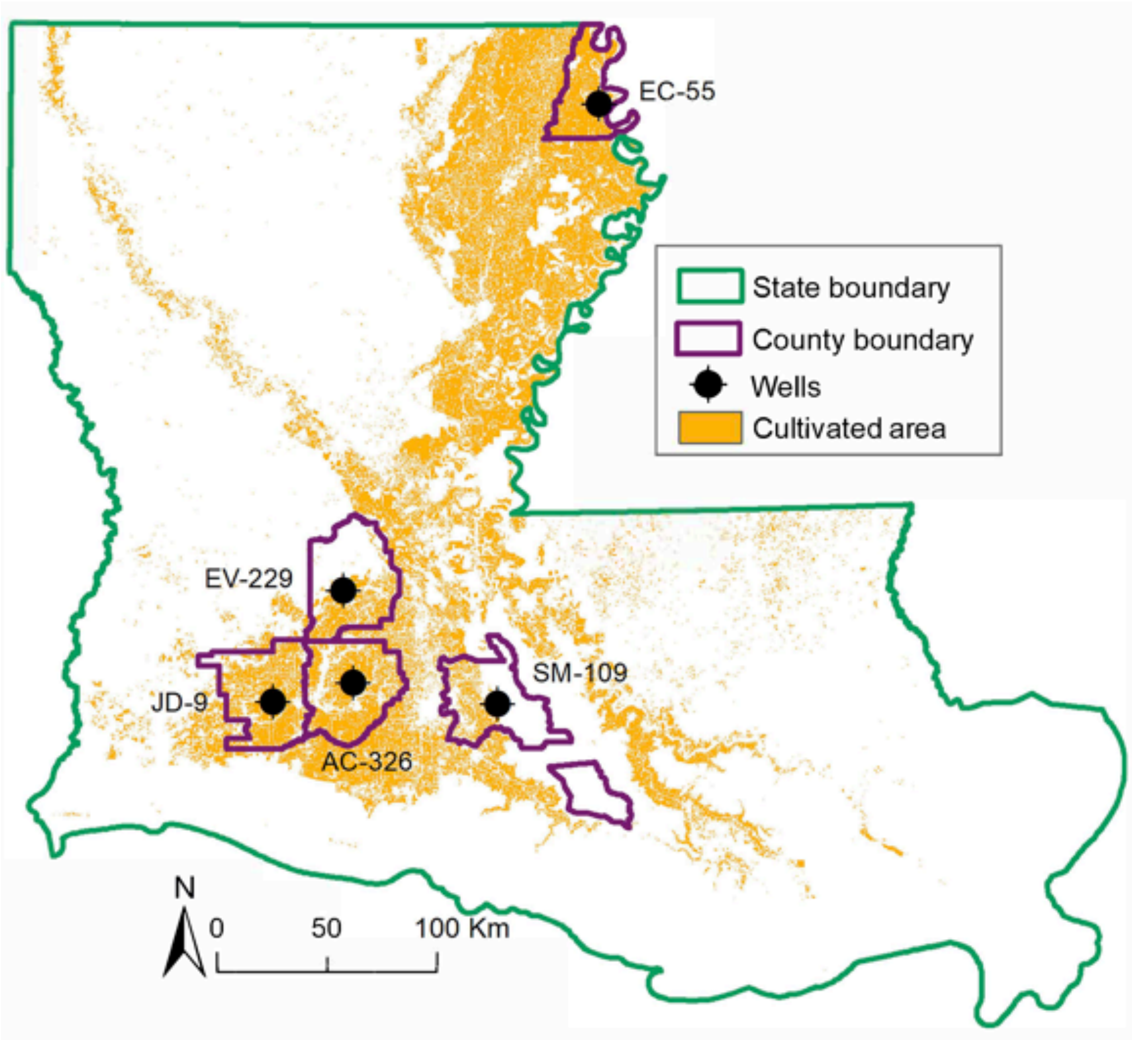

Figure S1 The locations of wells used in this study and agricultural area under cultivation along with the relevant county (parish) and state boundaries. Parishes: Evangeline (EV), Jefferson Davis (JD). St. Martin (SM), East Carroll (EC), Acadia (AC).
